# Self-assembled InAs/GaAs single quantum dots with suppressed InGaAs wetting layer states and low excitonic fine structure splitting for quantum memory

**DOI:** 10.1515/nanoph-2022-0120

**Published:** 2022-05-25

**Authors:** Xiaoying Huang, Jiawei Yang, Changkun Song, Mujie Rao, Ying Yu, Siyuan Yu

**Affiliations:** State Key Laboratory of Optoelectronic Materials and Technologies, School of Electronics and Information Technology, Sun Yat-Sen University, Guangzhou 510275, China

**Keywords:** fine structure splitting, self-assembled single QD, two photon excitation, wetting layer state

## Abstract

Epitaxial semiconductor quantum dots (QDs) have been demonstrated as on-demand entangled photon sources through biexciton–exciton (XX-X) cascaded radiative processes. However, perfect entangled photon emitters at the specific wavelengths of 880 nm or 980 nm, that are important for heralded entanglement distribution by absorptive quantum memories, remain a significant challenge. We successfully extend the QD emission wavelength to 880 nm via capping Stranski–Krastanow grown In(Ga)As/GaAs QDs with an ultra-thin Al_*x*_Ga_1−*x*_As layer. After carefully investigating the mechanisms governing the vanishing of wetting-layer (WL) states and the anisotropy of QDs, we optimize the growth conditions and achieve a strong suppression of the WL emission as well as a measured minor fine structure splitting of only ∼(3.2 ± 0.25) μeV for the exciton line. We further extend this method to fabricate In(Ga)As QDs emitted at 980 nm via introducing InGaAs capping layer, and demonstrate a two-photon resonant excitation of the biexciton without any additional optical or electrical stabilized source. These QDs with high symmetry and stability represent a highly promising platform for the generation of polarization entanglement and experiments on the interaction of photons from dissimilar sources, such as rare-earth-ion-doped crystals for solid quantum memory.

## 1 Introduction

Entangled photon pairs are one of the key elements for foundational quantum physics [1] and many emerging applications in photonic quantum information processing such as quantum computation [2], quantum key distribution [3], quantum repeater [4] and quantum metrology [5], etc. An ideal source of entangled photons requires a high degree of photon efficiency, photon indistinguishability and entanglement fidelity [6, 7]. Among different platforms, epitaxial semiconductor quantum dots (QDs) embedded in photonic structures have been successfully demonstrated as an on-demand entangled photon sources through biexciton–exciton (XX-X) cascaded radiative processes [8]. Pioneering works by Jin Liu et al. [9] and Hui Wang et al. [10] have demonstrated entangled photon pairs generated from GaAs/AlGaAs and InAs/GaAs QDs in circular Bragg resonators (CBRs), with a pair collection probability of large than 0.6, entanglement fidelity of ∼0.9, and indistinguishability of ∼0.9. However, there is still a lack of reports on the fabrication of perfect entangled photon emitters in other spectral ranges, such as 880 nm or 980 nm, which are important for heralded entanglement distribution by absorptive quantum memories based on rare-earth-ion-doped crystals [11, 12].

For InAs/GaAs QDs grown by Stranski–Krastanow (S–K) mode, increasing the size of QD islands [13] or changing the capping layer with InGaAs [14], are the straight strategies to extend the emission wavelength to 980 nm. However, the two-dimensional InGaAs wetting layer (WL), an inherent feature of the S–K mode, limits applications of QDs at 880 nm where the QD-WL energy gap is too small [15]. Moreover, the WL states often induces Coulomb interactions with QD charge carriers and therefore leads to hybridized quantum states, making the QD not an ideal artificial atom [16]. To alleviate this problem, one feasible approach is to apply a monolayer of AlAs layer on the top of InAs/GaAs QDs [17, 18]. This small modification can have profound impact on the photoluminescence properties of QDs [17], such as linewidth [19] and excitation efficiency [20]. The higher AlAs barrier can also blueshift the QD emission wavelength [21] to around 880 nm. To realize optimal entangled photon emitters, another challenge must be overcome is the fine structure splitting (FSS) of the neutral exciton state in asymmetric QDs. It is caused by atomistic asymmetry of the QD confining potential [22], which separates the two radiative decay paths and hence affects the time-averaged entanglement fidelity [23]. This issue has been addressed in other growth methods by developing highly symmetric QDs, including strain-free local-droplet-epitaxial GaAs QDs [24, 25], droplet InAs/GaAs or InAs/InP QDs on (111)A [26] with C_3v_ crystal symmetry, InGaAs grown on pre-template of inverted wet-etched pyramids [27], strain-controlled or modification of QDs [28] etc. However, for S–K InAs QDs, shape elongation easily occurs due to material intermixing and strain-related effects [29].

Here we take a further step towards entangled photon pairs emitted at 880 nm via S–K QDs by depositing a thin layer of Al_*x*_Ga_1−*x*_As on the top of InAs/GaAs QDs. Thanks to the large bandgap of Al_*x*_Ga_1−*x*_As, the electron WL-states are suppressed. Furthermore, the QD FSS was found to be directly correlated to the aluminum (Al) component due to the weak mobility of Al atoms but large mobility of indium atoms during indium flushing process. By removing the indium flushing process, symmetric 880 nm InAs QDs capped with Al_0.2_Ga_0.8_As can achieve an average FSS value of (9.4 ± 2.08) μeV among 30 single dots. The most symmetric QD demonstrates an FSS of only ∼(3.2 ± 0.25) μeV for the exciton line. This method was further extended to fabricate In(Ga)As QDs emitted at 980 nm via introducing InGaAs capping layer, which matches the absorption line of the ^171^Yb:Y_2_SiO_5_ crystal (978.854 nm) and therefore could be applied in solid-state quantum memory [12].

## 2 Material growth

The investigated samples were grown on semi-insulating GaAs (001) substrate in a solid source molecular beam epitaxy (MBE) chamber equipped with an arsenic (As) cracker cell and high-energy electron diffraction (RHEED). In brief, after growth of 300 nm GaAs buffer layer at the substrate temperature of 660 °C, InAs QDs were deposited at the temperature of (*Tc*-25) °C with an indium flux rate of 0.004 ML/s and an As flux pressure of 5 × 10^−7^ Torr. The deposition temperatures are calibrated by the transition temperature *Tc* when the surface reconstruction pattern of GaAs in RHEED transfers from (2 × 4) to (2 × 3) [30]. Then the InAs QDs were capped with a thin layer of 0.5 nm Al_*x*_Ga_1−*x*_As (*x* = 0, 0.2, 0.4, 0.6, 0.8, 1 for samples A–F) and subsequently over-grown by a layer of 6 nm GaAs, following by an indium flushing step [31] at the temperature of 660 °C. Finally, a layer of 100 nm GaAs was over-grown on the top of the QDs. Detailed growth parameters can be seen in Supplementary materials Table S1. A repeat layer of InAs QDs was deposited on the top of the GaAs layer without capping for further atomic force microscope (AFM) analysis, which was displayed in Supplementary materials Figure S1.

## 3 Results and discussion

**Figure 1:**
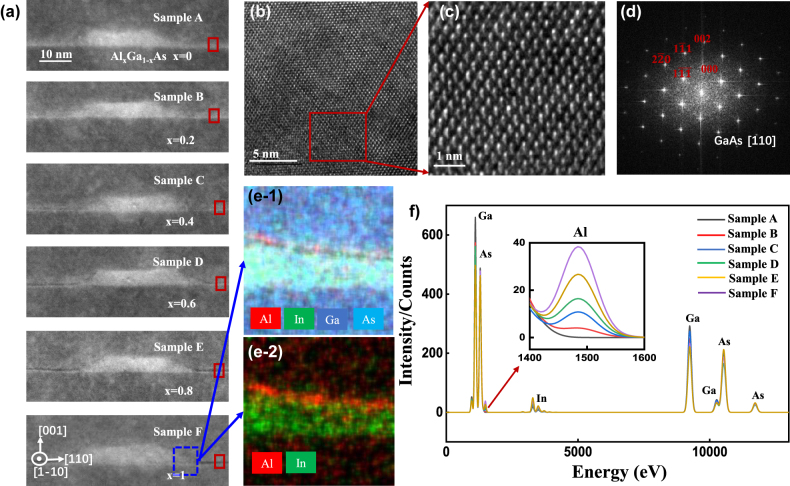
Morphologic properties of samples A–F. (a) STEM images taken along the [110] crystallographic direction of samples A–F, respectively. (b and c) High-resolution TEM images of the GaAs region. (d) The selected-area diffraction patterns in (c). (e) EDX elemental mapping of the blue box in sample F. (f) EDX element quantitative measurement of samples A–F with the same area of 3.5 × 3.5 nm from the red boxes in (a).

To determine the effect of Al_*x*_Ga_1−*x*_As capping layer on the morphology of InAs/GaAs QDs, scanning transmission electron microscopy (STEM) was carried out in a FEI Titan G2 60-300 Cubed at 300 kV equipped with an energy dispersive X-ray (EDX) microanalysis. Figure 1(a) shows the high-resolution high-angle annular dark field (HAADF) STEM images along [110] crystallographic direction of single dots from samples A–F. The bright regions close to the center of the image represent the InAs QDs, with the average height of ∼6 nm. The Al_*x*_Ga_1−*x*_As capping layer can be clearly presented as the darker region surrounding the QD. HRTEM images of GaAs material of sample F shown in Figure 1(b and c) illustrate the fine atomic arrangement, indicating high-quality and defect-free crystal structure. The diffraction pattern of the selected area displayed in Figure 1(d) confirms the crystal phase along [110] direction of GaAs [32]. Figure 1(e-1) and (e-2) illustrate EDX mapping images in the blue-dotted box of sample F, which clearly verifies the WL composition: 0.5 nm AlAs on top of ∼1 nm InGaAs WL. Further element quantitative measurement of samples A–F with the same area of 3.5 × 3.5 nm (red boxes in Figure 1(a)) is performed in Figure 1(f). The inserted image demonstrates that the concentration of Al in the WL increases with the Al component of Al_*x*_Ga_1−*x*_As capping layer of samples A–F.

**Figure 2:**
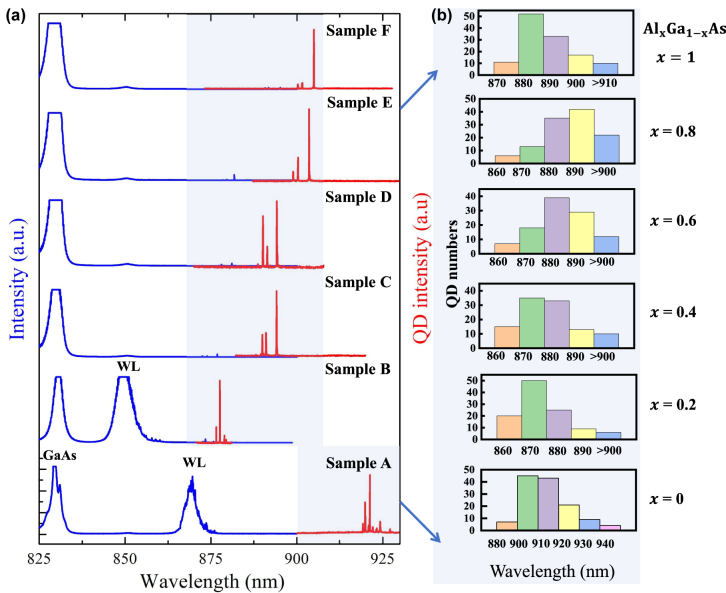
Optical properties of samples A–F. (a) Photoluminescence spectra of samples A–F with emission wavelength ranging from 825–930 nm (blue lines), corresponding representative PL spectra of Al_*x*_Ga_1−*x*_As-capped single QDs (red lines). (b) Wavelength distribution of about 100 single QDs of samples A–F.

Next we investigated the optical properties of InAs single QDs (SQDs) with Al_*x*_Ga_1−*x*_As capping layer, in which the suppressed WL emission is expected. The micro-photoluminescence (micro-PL) spectroscopy measurement is taken at the temperature of 4 K in a cryostat (Montana Instrument S100) under above-band excitation using 785 nm laser. As shown in blue lines of Figure 2(a), there is a strong emission from the WL at ∼875 nm with the GaAs-capped SQDs. For the Al_*x*_Ga_1−*x*_As-capped SQDs, WL emission is not observed when *x* = 0.4, 0.6, 0.8, 1, while the emission is blue-shifted to ∼850 nm when *x* = 0.2. It is worth mentioning that the weak emission at 850 nm in samples C–F is associated with the carbon-doping GaAs [33]. Red lines of Figure 2(a) illustrate typical PL properties of SQDs in samples A–F, together with the wavelength distribution of ∼100 SQDs in Figure 2(b). Compared with sample A with GaAs capping layer, the wavelength of Al_*x*_Ga_1−*x*_As-capped SQDs is blue-shifted from ∼910 nm [30] to ∼880 nm. It is remarkable that this kind of Al_*x*_Ga_1−*x*_As (*x* ≠ 0) capped SQDs shows distinctive three-exciton-line or four-exciton-line PL spectra, with deterministic binding energies.

**Figure 3:**
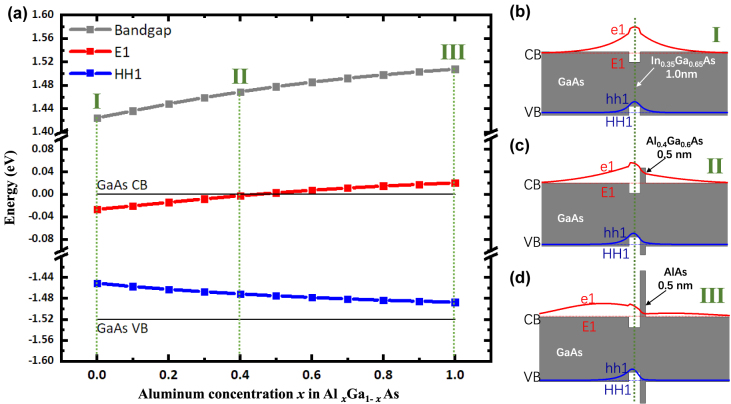
Calculated energy-band profiles for the GaAs/WL (1 nm)/Al_*x*_Ga_1−*x*_As/GaAs quantum wells (QW). (a) First excited state energies of electron (E1, red line) and heavy-hole (HH1, blue line), as well as the transition energy (E1→HH1, bandgap of WL, grey line) as a function of the aluminum concentration *x* in Al_*x*_Ga_1−*x*_As. (b–d) The first excited energy diagram and corresponding electron/hole wavefunctions where *x* = 0 (b), *x* = 0.4 (c), *x* = 1(d).

To reveal the essential mechanism of the vanishing emission of WL states, energy band structure for the GaAs/WL/Al_*x*_Ga_1−*x*_As/GaAs quantum wells (QW), and the corresponding electron/hole wavefunctions (e1/hh1) are evaluated using eight-band k·p method (Nextnano) [34, 35], as shown in Figure 3. It is found that the Al_*x*_Ga_1−*x*_As barrier greatly raises the electronic wavefunction and decreases the overlap of electron and hole’s wavefunctions. Thus the WL emission blue-shifts and the carrier confinement of WL becomes weak or even disappeared (*x* > 0.4) when increasing Al component in the Al_*x*_Ga_1−*x*_As capping layer. Moreover, since the radiative recombination coefficient is proportional to the square of the wavefunction overlap, a lower radiative recombination rate in WL with Al_*x*_Ga_1−*x*_As capping layer is also observed. Hence the ultra-thin Al_*x*_Ga_1−*x*_As capping layer can effectively suppress the WL states and yielding a better protection of the QD-electron and hole states from coupling to the WL than traditional GaAs capping layer.

**Figure 4:**
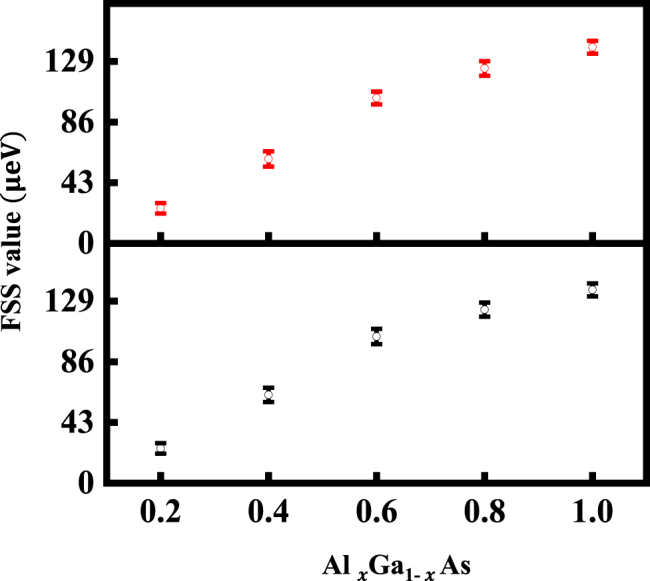
Average FSS values in samples B–F. The average FSS values of 30 SQDs in samples B–F, which increase as a function of Al component in Al_*x*_Ga_1−*x*_As capping layer. The black and red dots represent X_FSS_ and XX_FSS_, respectively.

We further measured the FSS values of neutral excitons and biexcitons of samples B–F using polarization-resolved micro-PL spectroscopy. The linear polarization of the emission was analyzed by a rotatable *λ*/2 achromatic retarder and a linear polarizer, dispersed by a spectrometer with a spectral resolution of ∼30 μeV. The relative peak positions are extracted from Lorentzian fits of the polarization-resolved micro-PL spectra of the representative SQDs and plotted as a function of the polarization angle. The average FSS value of 30 SQDs in each sample are presented in Figure 4. Representative FSS values of samples B–F are seen in the Supplementary materials Figure S2. It is found that the FSS values from the Al_*x*_Ga_1−*x*_As-capped QDs are strikingly different, which increase almost linearly as a function of the Al component in Al_*x*_Ga_1−*x*_As capping layer and reach an ultra large value of ∼140 μeV at *x* = 1.

We note that the FSS of In(Ga)As SQDs is associated with the strain/material inhomogeneity and shape elongation [29]. To investigate the formation mechanisms of the ultra-large FSS, we carefully analyzed the morphology of SQDs in Figure 1(a). We found that the average tilt angles of six single QDs from samples A–F among [110] crystallographic direction are 0.3°, 1.9°, 3.8°, 5°, 6°, 6.5°, respectively (Seen detailed statistical calculation in STEM images in Supplementary materials Figure S3). It is interesting to find that the SQD tilt direction is randomly distributed and its tilt angle increases almost as a function of Al component of Al_*x*_Ga_1−*x*_As in the capping layer. Similar inclination was observed in a previous study of single InAs QD capped by AlAs layer [17], yet the mechanisms were elusive. We consider both the anisotropy in the AlGaAs deposition and in the In(Ga)As desorption during indium flushing process contribute to the increase of FSS as a function of Al concentration in Al_*x*_Ga_1−*x*_As. A sketchy mechanism of the ultra-large FSS is depicted in Supplementary materials. The full theoretical explanation for the relation between the FSS value and the geometrical shape of tilted QDs is left for future investigations.

**Figure 5:**
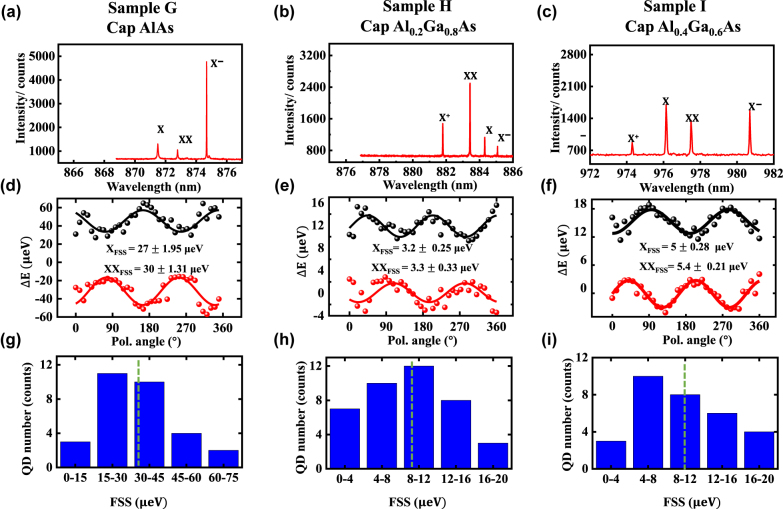
Optical properties of samples G–I. (a–c) Representative PL spectra of single QDs from samples G–I under above-band excitation; (d–f) the relative emission energy obtained by Lorentzian fits of the spectra in (a–c) as a function of polarization angles. The FSS values are obtained from the amplitude of a sine fitting. (g–i) The FSS histogram of X lines from samples G–I, the green dash lines represent measured mean FSS values of X line of each sample.

To confirm our speculation of the evolution in FSS values of Samples A–F, samples G and H were grown without indium flushing and capped with AlAs and Al_0.2_Ga_0.8_As, respectively. Representative PL spectra and corresponding wavelength distribution of SQDs are displayed in Supplementary materials Figure S5. As shown in the amplitude of the oscillations Figure 5(d), for sample G with AlAs capping layer, the typical FSS values for the X line are (27 ± 1.95) μeV. When decreased the Al component to *x* = 0.2 of Al_*x*_Ga_1−*x*_As capping layer in Sample H, the measured FSS values are significantly smaller: ∼(3.2 ± 0.25) μeV for the X line, as shown in Figure 5(e). Figure 5(g and h) summarize the statistics of FSS taken on ∼30 SQDs in both samples G and H. The results are predictable: the measured mean FSS value of sample G with AlAs capping layer ((32.2 ± 3.96) μeV for the X line) is larger than that of sample H with Al_0.2_Ga_0.8_As capping layer due to the higher Al component in the capping layer, which however is, as expected, much smaller than that of sample F with indium flushing process (∼140 μeV). Moreover, it is worth mentioning that the Al_0.2_GaAs capping layer prevents the InAs from intermixing with GaAs, which leads to an ultra-small mean FSS of (9.4 ± 2.08) μeV for the X line. However, the strain related effects are inevitable during the growth and maybe reduced through annealing [36] or the use of external electric/strain fields [37] in the future. We further applied this approach to the growth of sample I with emission wavelength ∼980 nm by capping a 4.5 nm In_0.2_Ga_0.8_As layer before growing Al_0.4_Ga_0.6_As capping layer (Detail growth parameters seen in Supplementary materials), which shows distinctive four-exciton-lines PL spectra (Figure 5(g)), with a measured mean FSS of ∼(10.2 ± 2.45) μeV for X line (Figure 5(i)).

**Figure 6:**
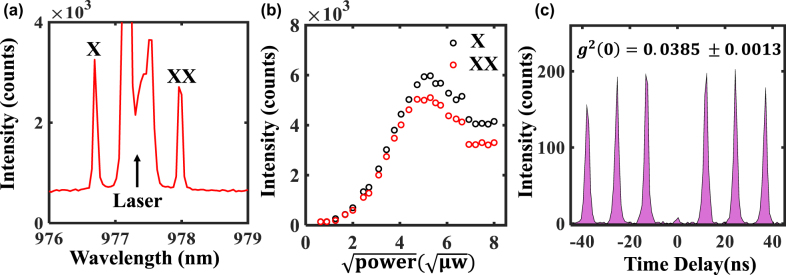
Two photon excitation of a 980 nm SQD on DBR. (a) Photoluminescence spectra of the biexciton–exciton cascade and a suppressed excitation laser. (b) Emission intensity of the exciton (black circles) and biexciton (red circles) lines under TPE as a function of the square root of the pump power, illustrating the damped Rabi oscillations. (c) Second-order autocorrelation measurement of g^(2)^(*t*) for typical QD exciton in (a).

To further demonstrate the potential of these entangled photon pair systems for quantum memory applications, measurements of the two-photon excitation of the biexciton were performed for a 980 nm single QD. To enhance the photon extraction efficiency, the SQD was embedded in a 2*λ*-GaAs layer on top of 40 pairs 1/4λ GaAs/Al_0.95_Ga_0.05_As distributed Bragg reflector (DBR) mirror. The excitation pulses were derived from a 76 MHz Ti: sapphire laser, whose temporal widths are adjusted by a pulse shaper consisting of two diffraction gratings and an adjustable slit placed in-between them. Figure 6(a) shows the TPE spectrum, obtained when the laser excitation energy is tuned exactly at the center of the XX and X energies. The spectrum displays two sharp peaks of X and XX lines, separated by a binding energy of 1.7 meV. Due to the stable QD states as well as the well confinement, photo-neutralization of charge environment using any off-resonant additional excitation source, such as a He–Ne laser [38] or a white light source [39] in the previous works, has been averted. Furthermore, to investigate the coherence of the two-photon–biexciton transition, we plot the integrated intensity of the biexciton and exciton in Figure 6(a) as a function of the square root of the excitation power. As can be seen in Figure 6(b), well resolved damped Rabi-type oscillations are observed, demonstrating a coherent nonlinear light–matter interaction [40]. Figure 6(c) shows the second-order autocorrelation measurement of X line, under ‘π-pulse’ two-photon resonant excitation by using a Hanbury–Brown and Twiss setup, revealing a g^(2)^ (*t* = 0) value of 0.0385 ± 0.0013. This result provides a clear evidence of the photon antibunching in the emission from these QDs.

## 4 Conclusions

In summary, we have demonstrated an effective QD growth method for generating entangled photon pairs at wavelength of 880 and 980 nm via capping S–K grown In(Ga)As/GaAs QDs with an ultra-thin Al_*x*_Ga_1−*x*_As layer. We have systematically investigated the influence of various Al component on the optical properties (exciton lines, FSS values) of In(Ga)As QDs. By decreasing the Al composition of AlGaAs capping layer and removing the indium flushing process, the WL states are strongly suppressed for our QDs. Extremely low FSS values less than 5 μeV are also achieved. The highly symmetric 880 nm (980 nm) QDs developed here can be valuable in the development of entanglement swapping and quantum memory for a quantum network and a quantum computer. In the near future, the photon extraction efficiency is expected to be significantly improved by embedding the QDs in broadband photonic structures, for example, circular Bragg grating [10], nanowire [41] or micrlens [42]. Large Purcell effect from circular Bragg grating cavity can also be helpful in efficiently mitigating the dephasing [43].

## Supplementary Material

Supplementary Material
