# Supplementary material for: Self-assembled InAs/GaAs single quantum dots with suppressed InGaAs wetting layer states and low excitonic fine structure splitting for quantum memory
Source: Nanophotonics. 2022 May 25;11(13):3093–100. doi: 10.1515/nanoph-2022-0120 (PMC11501422; doi:10.1515/nanoph-2022-0120)
Supplement: Supplementary file 1 — Supplementary Material [file j_nanoph-2022-0120_suppl.pdf]

Supplementary Materials

I. The detailed growth parameters of samples A-H

Table S1 Detailed QD growth parameters of samples A-H

Sample number	x in $Al_xGa_{1-x}As$	$Al_xGa_{1-x}As$ capping layer/nm	GaAs capping layer/nm	indium flux rate (ML/s)	Temperature (T_C-25℃)	As flux (Torr)
A	0	0.5	6 nm	0.004	575	5×10^7
B	0.2	0.5	6 nm	0.004	575	5×10^7
C	0.4	0.5	6 nm	0.004	574	5×10^7
D	0.6	0.5	6 nm	0.004	575	5×10^7
E	0.8	0.5	6 nm	0.004	576	5×10^7
F	1	0.5	6 nm	0.004	575	5×10^7
G	1	0.5	20 nm	0.004	573	5×10^7
H	0.2	0.5	20 nm	0.004	576	5×10^7

II. Representative AFM image of uncapped InAs QDs

Atomic force microscope (AFM, Bruker Icon) under peak force tapping mode was used to illustrate the size and density of single QDs (SQDs). As shown in Figure S1, the SQDs with single lines emission at ~900 nm display an average height of 7-8 nm and a typical diameter of 30 nm. The density is lowered down to almost 1 dot/ μm^2.

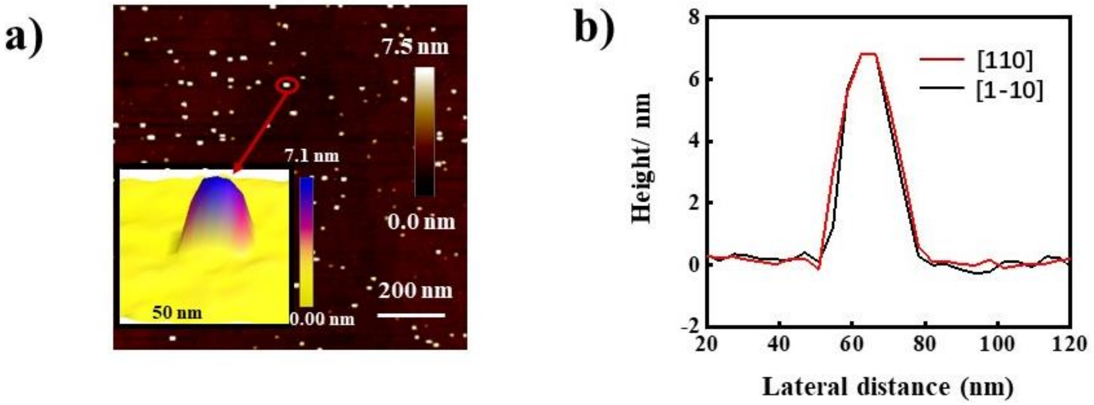

Figure S1. (a) Representative AFM image (1 μm × 1 μm) of single QD, the insert image shows the SQD size; (b) corresponding lateral profiles of the QD along [110] (red line) and [$1\bar{1}0$] (black line) crystallographic directions, respectively.

III. Representative FSS values of samples B-F

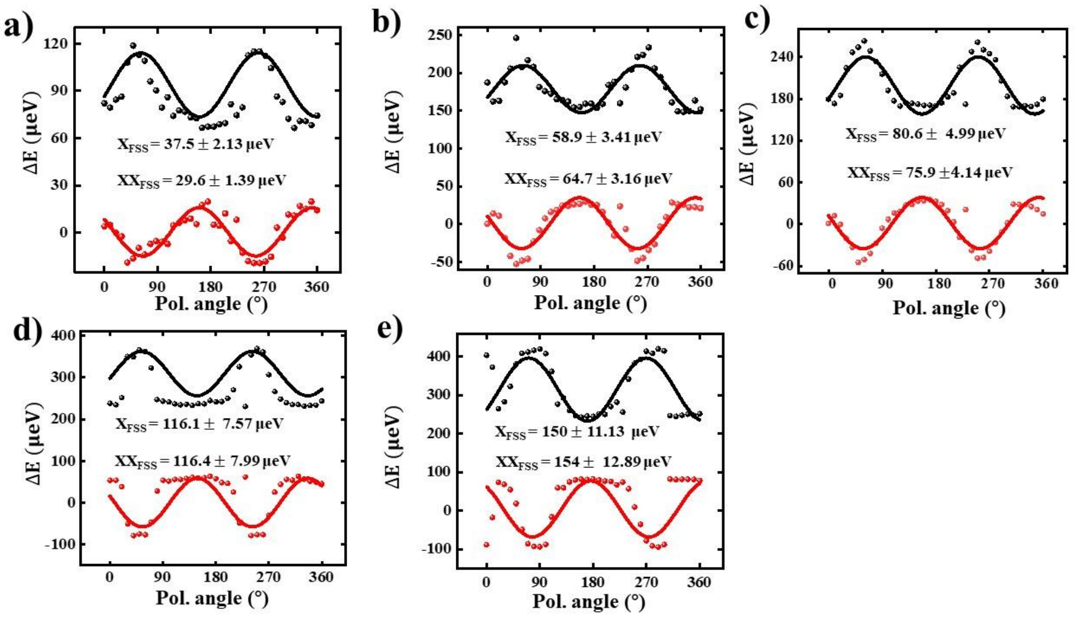

Figure S2. Representative FSS values of samples B-F in (a-e), respectively. The FSS value increases as a function of Al component in the capping layer.

IV. Representative STEM images of samples A-F

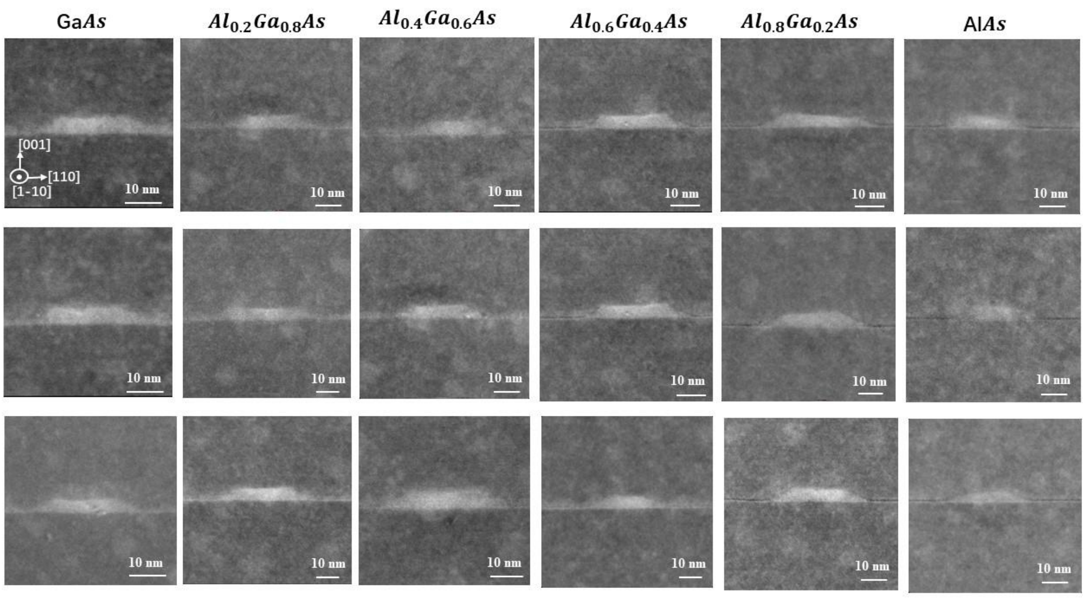

Figure S3. Three of representative SQD STEM images of samples A-F shows the tilt angle increases as the Al component in capping layer increase and the tilt direction randomly distributes in right and left.

V. The mechanism of the ultra-large FSS

To explain this phenomenon, another AlAs capped QD sample was grown without indium flushing process. A typical STEM image of SQD is displayed in Figure S4(a), revealing different thickness of AlAs on top of the SQD, which mainly caused by the weak mobility of Al atoms on the QD surface[1]. The similar phenomenon of non-uniform AlGaAs thickness could be observed in Samples B-F, as illustrated in Figure 4(b). Subsequently, when the substrate temperature was increased to the congruent evaporating temperature of InAs material during indium flushing process[2] (Figure 4(c)), the top of In(Ga)As QD beneath the ultra-thin $Al_xGa_{1-x}As$ layer became sufficiently active to break through the capping layer, and finally decomposed into In/Ga/Al and As atoms at the surface[3, 4]. The tilted QDs are supposed to form when meets the non-uniform thickness of $Al_xGa_{1-x}As$ layer. This obliquity in $[1\bar{1}0]$ crystallographic direction is larger than that of [110], as shown in Figures 4(e), owing to the higher desorption rate of facets on $[1\bar{1}0]$ (As-terminated staircase steps) compared with [110] (Ga-terminated staircase steps) as the activation barrier of $[1\bar{1}0]$ is smaller than [110][5, 6]. All these factors together we consider eventually contribute to the increase of FSS as a function of Al concentration in $Al_xGa_{1-x}As$.

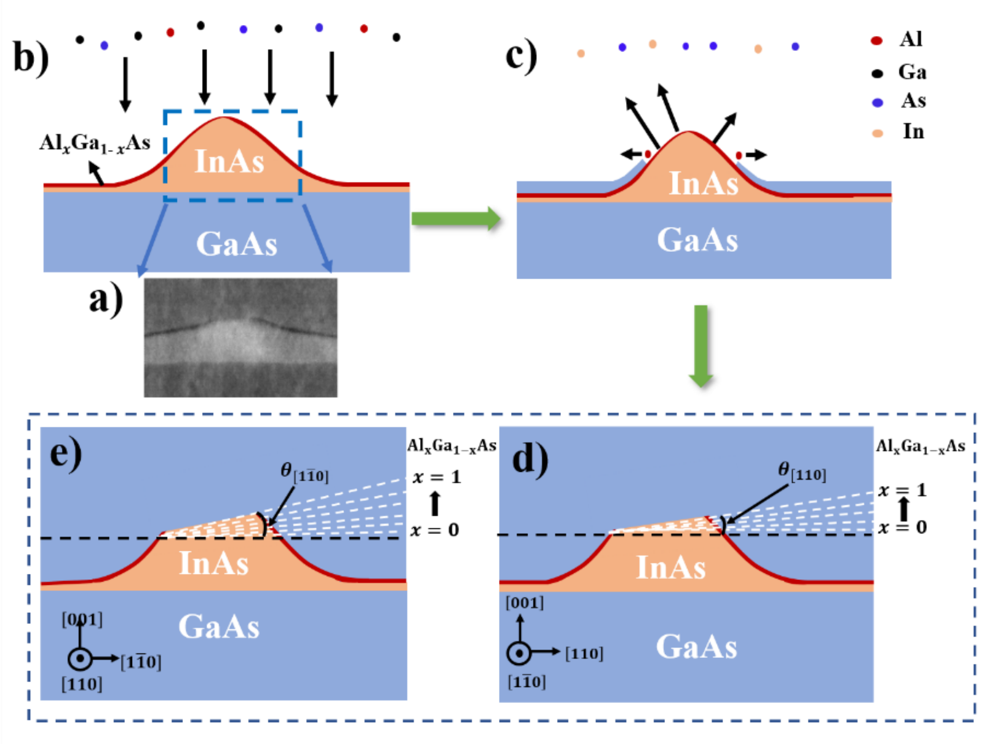

Figure S4: The illustration of the mechanism of the ultra-large FSS. (a) the STEM image of single QD capped with 0.5 nm AlAs without indium flushing. (b) deposition of 0.5 nm $Al_xGa_{1-x}As$ on the top of QD; (c) indium flushing process after 6 nm GaAs capping; (d-e) illustrate the randomly-oblique QD during indium flushing with $Al_xGa_{1-x}As$ capping, $\theta_{1\bar{1}0}$ and θ_{110} represent the tilt angle of QD in along $[1\bar{1}0]$ and [110] crystallographic directions, respectively. The tilt angles are achieved by statistically analyzing the STEM images in supplementary materials Figure S2.

VI. Optical properties of samples G and H

Figure S5 shows the representative photoluminescence (PL) spectra of WL and SQDs in sample G and sample H, respectively. The WL emission is not observed in sample G while it's blue shifted to 850 nm of sample H, which share the same PL properties with In-flushing QD of samples F and B, respectively.

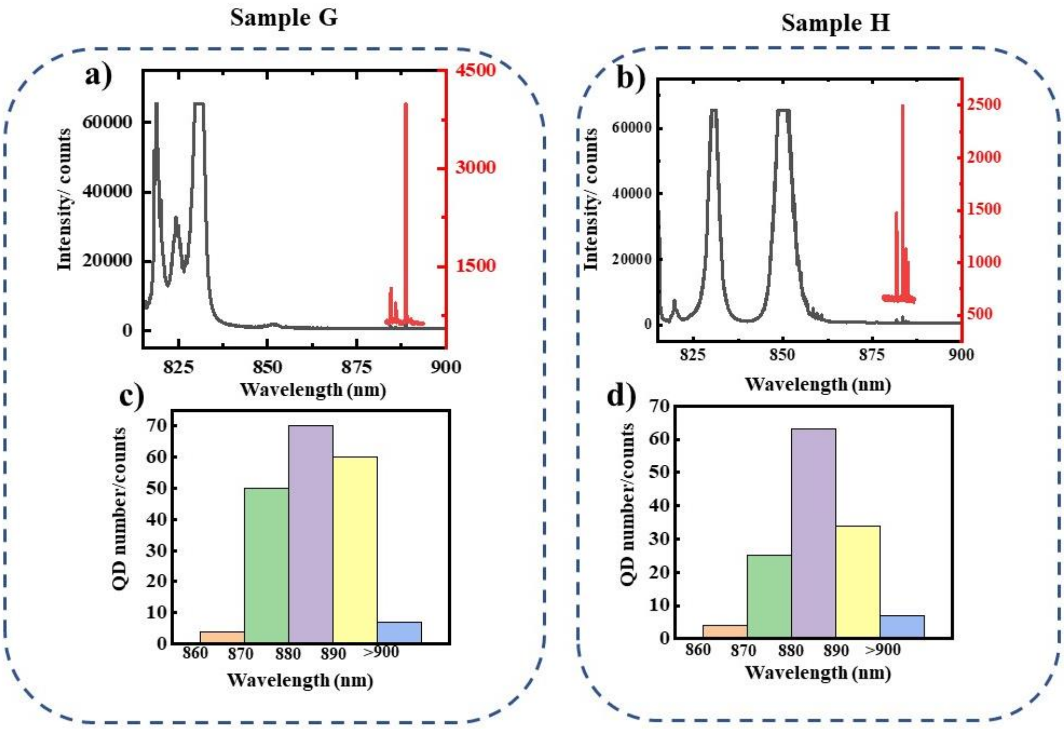

Figure S5. (a-b) Representative PL spectra of sample G and H with emission wavelength ranging from 820 nm to 900 nm as shown in black lines, the red lines represent the PL of SQDs, together with corresponding wavelength distribution of SQDs in (c-d), respectively.

VII. Growth method and optical properties of SQDs emitted at 980 nm

Growth Method

After growth of 300 nm GaAs buffer layer, InAs QDs were deposited at the temperature of (Tc-25)℃ with an indium (In) flux rate of 0.004 ML/s and an arsenic (As) flux pressure of 5×10^{-7} Torr. Then 4.5 nm $In_{0.15}Ga_{0.85}As$ strain reduce layer (SRL) was deposited on the top of InAs QDs with In deposition rate of 0.2 ML/s, Ga deposition rate of 1 ML/s and As flux pressure of 1.9×10^{-5}Torr. Finally, 0.5 nm $Al_{0.4}Ga_{0.6}As$ and 100nm GaAs were overgrown on the top of InGaAs SRL.

Optical Properties

Figure S6 shows PL spectra of SQDs with InGaAs SRL and $Al_xGa_{1-x}As$ capping layer (x=0, 0.4, 1). The emission at 930 nm represents GaAs/InGaAs/GaAs quantum well and the emissions at ~980 nm represents SQDs. The properties of SQDs change obviously in different Al composition in the capping layer. $Al_xGa_{1-x}As$-capped SQDs show distinctive three-line and four-line spectra, which is the same as InAs QDs emitted at 880 nm.

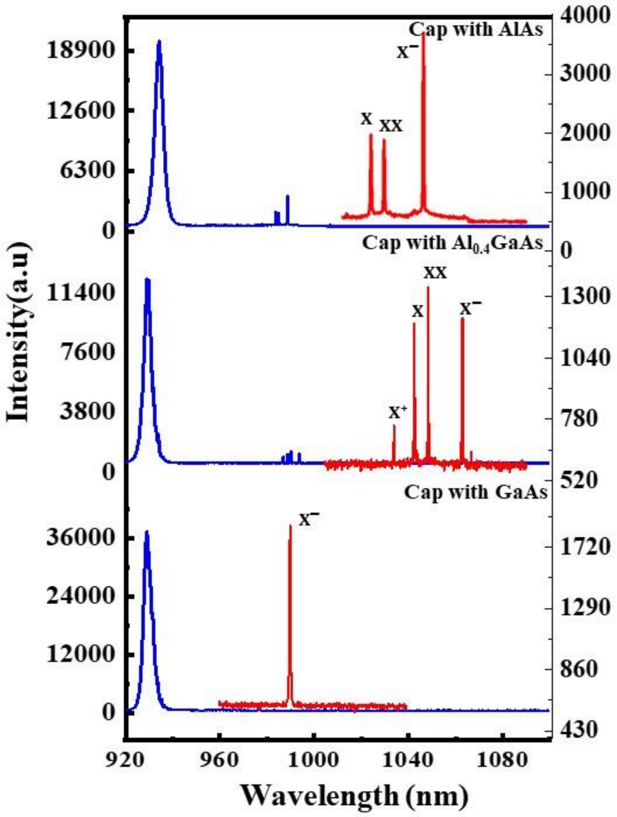

Figure S6. Black lines show ensemble spectra with emission wavelength ranging from 920-1100 nm and red lines show corresponding PL spectra of SQDs emitted at ~980 nm with $Al_xGa_{1-x}As$ capping layer (x=0, 0.4, 1).

1. Saito, J.; Kondo, K., High-temperature growth of Si-doped AlGaAs by molecular-beam epitaxy. *Journal of Vacuum Science & Technology B: Microelectronics Processing and Phenomena* **1990,** *8* (6), 1264-1269.
2. Yang, H. J.; Choi, S.; Kim, J. H.; Kim, I.; Ahn, S. J.; Lee, H. S.; Yi, S. H.; Kim, Y. H., Thermodynamic patterns duringin-situheating of InAs nanowires encapsulated in Al2O3shells. *Nanotechnology* **2021,** *33* (2).
3. Manasson, A.; Bah, M.; Desmarais, B.; Douglass, D.; Outten, C.; Schumacher, J.; Robinson, M.; Zhang, C., *Indium bump deposition for flip-chip micro-array image sensing and display applications*. SPIE: 2018; Vol. 10639.
4. Alonso-Álvarez, D.; Alén, B.; Ripalda, J. M.; Rivera, A.; Taboada, A. G.; Llorens, J. M.; González, Y.; González, L.; Briones, F., Strain driven migration of In during the growth of InAs/GaAs quantum posts. *APL Materials* **2013,** *1* (2), 022112.
5. LaBella, V. P.; Bullock, D. W.; Ding, Z.; Emery, C.; Harter, W. G.; Thibado, P. M., Monte Carlo derived diffusion parameters for Ga on the GaAs(001)- (2×4) surface: A molecular beam epitaxy–scanning tunneling microscopy study. *Journal of Vacuum Science & Technology A* **2000,** *18* (4), 1526-1531.
6. Shitara, T.; Zhang, J.; Neave, J. H.; Joyce, B. A., Ga adatom incorporation kinetics at steps on vicinal GaAs (001) surfaces during growth of GaAs by molecular beam epitaxy. *Journal of Applied Physics* **1992,** *71* (9), 4299-4304.
